# Using Ecological Momentary Assessment to Study Tobacco Behavior in Urban India: There’s an App for That

**DOI:** 10.2196/resprot.4408

**Published:** 2015-06-24

**Authors:** Andrea Soong, Julia Cen Chen, Dina LG Borzekowski

**Affiliations:** ^1^ Institute for Global Tobacco Control Department of Health, Behavior & Society Johns Hopkins Bloomberg School of Public Health Baltimore, MD United States; ^2^ School of Public Health Department of Behavioral and Community Health University of Maryland College Park College Park, MD United States

**Keywords:** ecological momentary assessment, tobacco control, cell phones, mobile phones, mHealth, telemedicine, smoking

## Abstract

**Background:**

Ecological momentary assessment (EMA) uses real-time data collection to assess participants’ behaviors and environments. This paper explores the strengths and limitations of using EMA to examine social and environmental exposure to tobacco in urban India among older adolescents and adults.

**Objective:**

Objectives of this study were (1) to describe the methods used in an EMA study of tobacco use in urban India using a mobile phone app for data collection, (2) to determine the feasibility of using EMA in the chosen setting by drawing on participant completion and compliance rates with the study protocol, and (3) to provide recommendations on implementing mobile phone EMA research in India and other low- and middle-income countries.

**Methods:**

Via mobile phones and the Internet, this study used two EMA surveys: (1) a momentary survey, sent multiple times per day at random to participants, which asked about their real-time tobacco use (smoked and smokeless) and exposure to pro- and antitobacco messaging in their location, and 2) an end-of-day survey sent at the end of each study day. Trained participants, from Hyderabad and Kolkata, India, reported on their social and environmental exposure to tobacco over 10 consecutive days. This feasibility study examined participant compliance, exploring factors related to the successful completion of surveys and the validity of EMA data.

**Results:**

The sample included 205 participants, the majority of whom were male (135/205, 65.9%). Almost half smoked less than daily (56/205, 27.3%) or daily (43/205, 21.0%), and 4.4% (9/205) used smokeless tobacco products. Participants completed and returned 46.87% and 73.02% of momentary and end-of-day surveys, respectively. Significant predictors of momentary survey completion included employment and completion of end-of-day surveys. End-of-day survey completion was only significantly predicted by momentary survey completion.

**Conclusions:**

This first study of EMA in India offers promising results, although more research is needed on how to increase compliance. End-of-day survey completion, which has a lower research burden, may be the more appropriate approach to understanding behaviors such as tobacco use within vulnerable populations in challenging locations. Compliance may also be improved by increasing the number of study visits, compliance checks, or opportunities for retraining participants before and during data collection.

## Introduction

### Overview

This study describes the ecological momentary assessment (EMA) of tobacco use in urban India, using a mobile phone app for data collection. This exploration was the first step of a larger study examining overall use of, and exposure to, tobacco. In this paper, we focus on the feasibility of using EMA in the Indian cities of Hyderabad and Kolkata. To better understand tobacco use, particularly in low- and middle-income countries (LMIC) where usage is high [1,2], it is critical to use valid and reliable methods. Innovation and new technology may advance such work, but researchers must know the strengths and limitations of using such methods.

This paper begins by offering background information from the current EMA literature, which focuses mainly on work done in the United States and other developed countries. Next, we describe our design and protocols, done with a sample of 205 participants across India. Then, we review the EMA approach, drawing on participant completion and compliance rates. We conclude with general recommendations on implementing mobile phone EMA research in India and other LMIC.

### Background

Current and past surveillance of global tobacco use relies primarily on retrospective recall—in the last two decades, EMA methods have been proposed as a viable counterpart to traditional recall methods in health behavior research. EMA is broadly defined as a repeated real-time collection of data on subjects’ behavior and experience in their natural environments [3]. The use of multiple and brief—usually less than five minutes—assessments over a given period of time captures a representative experience of the participants’ environments. EMA data may “shed light on relationships that are missed when relying on retrospective self-reports” [3]. EMA can be applied to a wide range of behavioral and clinical psychology research, and these methods are particularly advantageous in studying discrete and episodic behaviors, such as drug or substance use [4].

There is extensive EMA research in the United States on cues to smoke cigarettes among participants enrolled in cessation programs [5-8], as well as several studies of youth exposure to protobacco media [9,10]. In focusing primarily on regular smokers and cessation behaviors, EMA studies tend to offer implications for clinical and intervention advancements in substance abuse [4]. Studies by Martino, Shadel, and colleagues that consider adolescent and young adult social exposure to tobacco use and media often lead to discussions around advocacy and regulations [9,10].

In Japan, two EMA research studies using handheld computers have examined patient symptoms and clinical care. One study assessed aggravators of tension headaches and the other assessed symptoms experienced by home care hospice patients [11,12]. Both studies used compact computers as the EMA device and considered health behaviors among a disease-specific patient population.

Mobile technology health initiatives, also known as telehealth or mHealth, have occurred in LMIC and share some characteristics with EMA with respect to the use of technology. The difference, however, concerns research aims. The term mHealth refers to the “delivery of, and access to, health services and information” via mobile technologies [13]. While EMA employs mobile technology and can certainly fit under this definition, there are distinctions. A 2013 review of mHealth projects among the US clinical federal trial registry, conducted by Labrique and colleagues, identified over 90% of the work as “interventional” rather than “observational” [14]. Indeed, the primary focus of domestic and international mhealth research usually pertains to clinical or behavioral interventions, such as increasing treatment adherence via text message reminders, tracking and monitoring vitals, or facilitating communication between providers and patients [13]. EMA, in contrast, takes a more observational approach, assessing momentary events of a particular phenomenon as it is experienced by participants in the natural environment.

### India and Ecological Momentary Assessment

To date, we are unaware of any EMA studies of tobacco cues in India, or for that matter, in any LMIC. India is a country with rates of high tobacco use as well as high mobile phone penetration, making it a strong fit for an EMA tobacco study. According to the World Health Organization (WHO) Global Adult Tobacco Survey (GATS) data from 2009, overall tobacco use in any form was nearly 50% for males and 20.3% for females in India [1]. Adolescent tobacco use is prevalent, and becoming more equally distributed between genders [15,16]. Surrogate advertising, or tobacco brand extension through nontobacco products, such as clothing or food, is a common method of increasing tobacco sales in India, even though it violates local and national legislation [17-19]. Point-of-sale tobacco advertising is largely unrestricted, and there is reportedly low success in preventing product sales to minors [18].

Amid a sharp worldwide increase of mobile phone penetration over the past decade, mobile phones are a practical way to collect real-time public health data. India had more mobile phone subscriptions in 2011 than Africa, the Middle East, and Europe—72 out of every 100 inhabitants in India have a mobile/cellular subscription [20]. Although advanced feature mobile phone users only make up 9% of all mobile users in India, the penetration is twice as high in urban areas [20]. Adults aged 18 to 24 years also make up a higher-than-average proportion of mobile phone users at 13% [20].

## Methods

### Surveys and Data Administration

A team of researchers beta tested early versions of the study protocol and EMA app in February 2013 in Hyderabad, India. Data collection was completed from February to May 2014 in staggered time periods, in Hyderabad and Kolkata, India. Ethics approval was obtained from the Johns Hopkins University Institutional Review Board (IRB), University of Maryland College Park IRB, and the BioMedical Ethics Committee in India. The project was supported by an award from the Institute for Global Tobacco Control at the Johns Hopkins Bloomberg School of Public Health with funding from the Bloomberg Initiative to Reduce Tobacco Use.

Prior to instrument and protocol development, the team conducted in-depth interviews with EMA field experts, as well as a series of focus groups to gain insight into the feasibility of conducting EMA research in a foreign setting using mobile phones. We contracted a local public health research company based in Hyderabad, India, to act as an in-country partner to assist with survey translation, field staff training, participant recruitment, and in hiring a local developer to create the EMA data collection app.

**Figure 1:**
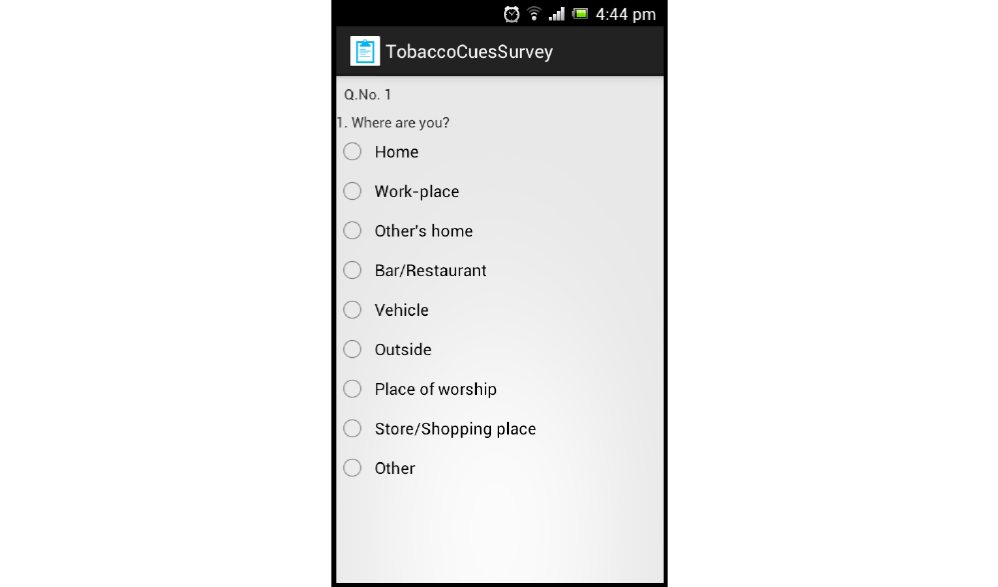
Screenshot of the ecological momentary assessment (EMA) mobile app.

For this type of study, the EMA literature supported a time-based sampling approach, in which the researcher determines the intervals and moments at which participants are prompted for data collection. This approach, as opposed to event-based monitoring, in which participants can manually initiate a survey on their own, was a better fit because “time-based sampling typically aims to characterize experience more broadly and inclusively...without a predefined focus on discrete events” [3]. The developed EMA app consisted of two surveys. The first EMA survey was a momentary survey, consisting of multiple-choice questions adapted from EMA questionnaires typically done on personal digital assistants (PDAs). Questions came from the EMA literature [5] and adapted portions of the GATS India [21]. Momentary prompts asked participants to give their general location (eg, at home, in a restaurant), social setting (eg, alone, with friends), and tobacco environment (eg, do they see smokers or tobacco advertisements?). Figure 1 presents a screenshot of the first question of the momentary survey. We excluded fill-in responses in the momentary survey to maximize efficiency for survey completion time as well as data analysis, as per recommendations from an EMA field consultant (personal communication, S Shiffman, August 2012).

The second EMA survey was an end-of-day (EOD) survey that recorded the participants’ tobacco use and observations from the previous 24-hour period. Although these types of questions required a brief period of recall, such daily diaries are considered a lower-burden form of EMA, and can be compared with momentary EMA data to determine whether it is a suitable proxy [3,22]. The end-of-day survey included a fill-in response to capture qualitative data in case the participant had a memorable or unique observation of the day’s events. We did not set a limit on word count because we expected answers to be relatively brief. The complete momentary and end-of-day questions and response options are located in Multimedia Appendix 1 (sections A and B).

We also developed and employed a traditional paper-and-pencil baseline survey to be administered prior to app installment and training. This survey asked participants about demographic information, tobacco use, and perceptions. All surveys were written below an 8th-grade reading level to increase accessibility to lower-literacy participants.

No personal identifiers appeared in the data returned from the EMA surveys. We retained participant names and phone numbers during data collection for the purposes of compliance checks and follow-up, but these data were not linked to their responses. The app automatically forwarded and stored data from completed momentary and end-of-day surveys to a private secure server owned and operated by the in-country team. The app did not require Internet connection for survey completion. This was an important feature since intermittent power outages are common in the study area. Internet access, however, was required to eventually forward EMA data to the server. The app recorded incomplete or timed-out surveys as "expired" in the dataset and did not record partial responses. If participants turned off their mobile phones or if phones were in a poor reception area, the app would mark data as missing or display blank cells in the dataset.

### Participant Recruitment and Training

Inclusion criteria for the study included possession of an Android-series mobile phone, or one with similar functions and app capabilities—several off-brand versions of the Android phone existed in the study site. Participants needed to be literate so that consent could be given, and so that one could read and complete surveys. The eligible age for participation was 16 to 40 years. The study did not require technological expertise with a mobile phone, as the research team provided extensive training on the app. Participants received a month of unlimited data for their phone upon enrollment in the study, as well as a flash drive when they completed the study.

Our in-country partner recruited participants from Hyderabad and Kolkata, India, through local schools and colleges, work offices, and popular neighborhood places, such as cafes, restaurants, and bars. Schools primarily consisted of local colleges and universities, but we included some high schools in the recruitment in order to enroll older adolescent participants. Participants 18 years of age and older provided written consent to join the study, while participants younger than 18 years were additionally required to provide parental consent as well as assent. After enrollment, participants completed the baseline survey and then research staff familiarized each participant with the study procedures. During this initial session, staff installed the EMA app onto the participants' mobile phones, and instructed participants on using the EMA app. They also discussed if and how various tobacco cues might be in the participant’s environment. Participants practiced using the EMA app, and staff answered questions or alleviated difficulties.

### Procedure

For 10 consecutive days in February 2014, the EMA app randomly signaled participants on their mobile phone five to eight times per day during waking hours—defined as 8am to 10pm—and prompted them to complete the momentary survey. Unless directed otherwise, the end-of-day survey prompt occurred at 10pm on each day of data collection. This sampling scheme resembled that of similar studies in the EMA tobacco literature [3,6,9]. Momentary and end-of-day prompts were designed to take 3 to 5 minutes and less than 5 minutes, respectively. Identical questions in the two surveys were used, though the order of momentary survey questions changed to prevent response fatigue. Participants had 30 minutes to complete a survey once they opened the app and started a survey—if they did not finish in time, the dataset would show the prompt as expired. If participants were unable to use their phone when signaled, for example, during a meeting or while driving, they could put the EMA app on hold or "snooze" for up to 20 minutes. During this "snooze" time, the app would signal every 5 minutes to remind them to take the survey. After 20 minutes, the participant could no longer take that particular survey and it would be recorded as expired. This feature was modeled from Shiffman’s work [6]. The app signaled users automatically—participants could not manually initiate or self-initiate a survey.

### Study Variables

The following section describes variables used in the baseline, momentary, and end-of-day surveys, although a separate paper considers the participants’ tobacco use and exposure to tobacco use and messaging (personal communication, DG Borzekowski and JC Chen, February 2015).

### Baseline Survey

Demographic variables included age, gender, employment, education level, and car ownership in the household. Participants’ current tobacco use status consisted of separate variables for the use of smoked and smokeless forms, respectively—not at all, smoke less than daily, and smoke daily; not at all, use less than daily, and use daily. We did not exclude tobacco users in the study because we wanted to be able to compare differences in tobacco exposure and messaging as experienced in the natural environment by tobacco use status. These data are examined in a separate paper by Borzekowski and Chen (personal communication, February 2015). The baseline measured exposure to pro- and antitobacco messages, respectively—ever saw information promoting tobacco use, ever saw information discouraging tobacco use—for various locations that participants may have visited over the past 30 days (eg, government building, hospital, school, workplace, public transportation).

### Momentary Survey

The momentary survey asked about participants’ current use of tobacco as well as their social and environmental exposure to tobacco. First, the survey considered the participants’ physical and social setting. Variables included location—home, workplace, other’s home, bar/restaurant, vehicle, outside, place of worship, store/shopping place, other—and social setting—alone or with others. Next, the survey considered participants’ momentary tobacco environment, with variables on personal use of tobacco—none, smoked, or smokeless—and tobacco use by other people nearby—none, in participant's social group, or in view. If the participant reported using or seeing others use tobacco, the survey generated additional variables for product type—brand name cigarette, rolled cigarette, bidi, cigar, cigarillo—and brand. Other environmental variables in the survey included observation—yes or no—of paraphernalia related to tobacco use, such as used ashtrays, butts, spit from oral tobacco, or smelling secondhand smoke. Lastly, variables related to tobacco media exposure included observation—yes or no—of pro- and antitobacco messages in their location appearing in various places—newspapers or magazines, television, radio, billboards or posters, or on cigarette or smokeless tobacco packs. Protobacco messages were defined as those promoting tobacco products and antitobacco messages were defined as those warning about the dangers of using tobacco or encouraging quitting.

### End-of-Day Survey

The end-of-day survey considered participants’ tobacco use that day—yes or no—with variables for product type—smoked or smokeless—and brand, if answered positively. Variables included observation—yes or no—of other people using tobacco that day and, if yes, who—friends, family/relatives, spouse, coworkers, or people they did not know. Variables on paraphernalia related to tobacco use—seeing ashtrays, butts, spit, or smelling secondhand smoke—were similar to those used in the momentary survey. Variables on exposure to pro- and antitobacco messages seen over the past day resembled those in the momentary survey, though the survey questions were structured slightly differently. A final measure asked participants to compare that day to others, indicating if they had witnessed higher, lower, or similar amounts of tobacco cues.

### Data Analysis

This study examined participant compliance data to assess and demonstrate the feasibility of using an EMA approach. To analyze the data, the researchers considered the following: (1) what variables, such as participants’ baseline characteristics, real-time tobacco use, and social and environmental cues, predicted the momentary and end-of-day compliance throughout the study period, and (2) the convergent validity of the daily end-of-day survey alongside the momentary surveys.

### Ecological Momentary Assessment Compliance Measurements

The measurable outcomes for examining participant compliance involved identifying the total number of momentary and end-of-day surveys received by participants, and identifying the total number of momentary and end-of-day surveys completed by participants. With this information, we then calculated the proportion of momentary and end-of-day surveys received and completed, which we refer to as the completion rates. We also considered the number of days in the 10-day study period within which the participants completed the momentary or end-of-day surveys, and the amount of time involved for participants to complete momentary and end-of-day entries.

### Statistical Analyses

We employed simple analyses to examine this dataset. To predict the factors influencing momentary and end-of-day compliance, we estimated two linear regressions. We used momentary completion rate as a dependent variable, and used the end-of-day completion rate, baseline characteristics, and other real-time tobacco-related measurements as independent variables. Similarly, in order to predict the factors that influence end-of-day compliance, we used the end-of-day compliance rate a dependent variable, and the same variables listed previously as independent variables. We also conducted zero-order correlation tests to analyze the relationship between momentary and end-of-day surveys, using outcome data from the first 5 and all 10 days of data collection.

## Results

### Sample Characteristics

The sample included 205 participants, of which 135 (65.9%) were male and 70 (34.1%) were female. The median age was 23 years (interquartile range [IQR] 9). Sample size for other baseline variables varied based on participant response—133 out of 202 respondents (65.8%) had attained less than a college degree, 147 out of 205 (71.7%) were unemployed, and 122 out of 200 (61.0%) owned a car. Most participants did not regularly use tobacco—out of 205, 106 (51.7%) smoked "not at all," 56 (27.3%) smoked "less than daily," and 43 (21.0%) smoked "daily." Smokeless tobacco use was extremely rare in the sample. Only 9 out of 205 participants (4.4%) reported any use of smokeless tobacco—7 (3.4%) used it "less than daily," and 2 (1.0%) used it "daily." All of the 9 smokeless tobacco users were dual users of smoked and smokeless products.

### Ecological Momentary Assessment Compliance Measurements

#### Momentary Survey

Participants received a total of 11,954 momentary surveys from the EMA app. Participants completed and returned a total of 5603 surveys—6351 surveys expired due to incomplete response or nonresponse.

**Figure 2:**
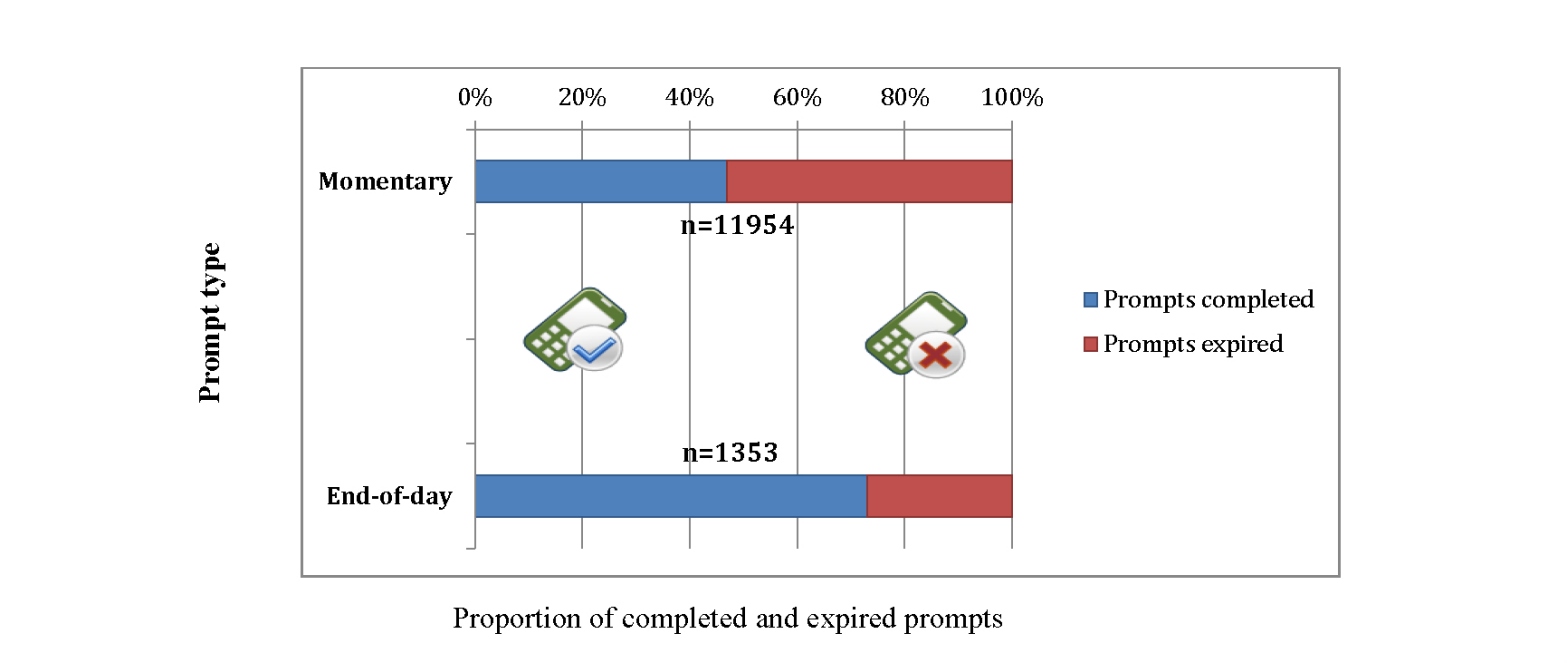
Comparison of completed and expired ecological momentary assessment (EMA) survey prompts, by type.

The completion rate was .47 (SD .21), or 46.87% (5603/11,954). Figure 2 gives a visual comparison between the completion rates of momentary and end-of-day assessments.

On average, participants completed surveys for 7.29 (SD 2.56) days out of the 10-day period, and spent 3.84 (SD 2.21) minutes to complete a single survey. A total of 39 out of 205 (19.0%) of the participants completed at least one momentary survey every day during the 10-day study period.

#### End-of-Day Survey

Participants received a total of 1353 end-of-day surveys from the EMA app. Participants completed and sent back a total of 988 end-of-day surveys—365 surveys expired due to incomplete response or nonresponse. The completion rate for the end-of-day surveys was .73 (SD .27), or 73.02% (988/1353). On average, participants completed surveys for 6.98 (SD 2.62) days out of the 10-day period, and spent 3.62 (SD 2.82) minutes to complete a single survey. A total of 54 participants out of 205 (26.3%) completed all of the end-of-day surveys over the 10-day study period.

### Ecological Momentary Assessment Compliance Predictors

#### Overview

Table 1 presents models predicting EMA and EOD completion rates. In the first model, a higher end-of-day completion rate (beta=0.11, 95% CI 0.05-0.15, *P*=.001), being employed (beta=0.10, 95% CI 0.02-0.17, *P*=.01), seeing other people using tobacco (beta=-0.17, 95% CI -0.27 to -0.05, *P*=.02), and not being exposed to messages discouraging tobacco use (beta=-0.15, 95% CI -0.29 to -0.02, *P*=.01) predicted a higher momentary completion rate. The overall model fit was *R* ^2^=.14 (*F*_14,173_= 3.1, n=188). This model explained about 14% of the overall outcome variance. In the second model, the momentary completion rate (beta=0.64, 95% CI 0.16-1.13, *P*=.01) strongly predicted the end-of-day completion rate. The overall model fit was *R* ^2^=.03 (*F*_14,167_=1.4, n=182). This model explained only 3% of the overall outcome variance. The relatively low *R* ^2^ of both models indicated that other unexplored factors might be associated with momentary and end-of-day compliance outcomes.

**Table 1:** Predictors of momentary and end-of-day study compliance.

Variable item		Momentary compliance^a^				End-of-day compliance^b^			
		beta	SE	*P*	95% CI	beta	SE	*P*	95% CI
Momentary compliance		N/A^c^	N/A	N/A	N/A	0.64	0.25	.01^d^	0.16 to 1.13
End-of-day compliance		0.12	0.03	.004	0.07 to 0.18	N/A	N/A	N/A	N/A
**Gender**									
	Male	Reference				Reference			
	Female	0.02	0.04	.61	-0.05 to 0.09	-0.20	0.12	.14	0.16 to 1.13
Age		0	0	.70	-0.01 to 0	0	0.01	.86	-0.002 to 0.12
Education		0.01	0.16	.54	-0.02 to 0.04	0.07	0.05	.23	-0.04 to 0.17
**Employment**									
	Unemployed	Reference				Reference			
	Employed	0.10	0.04	.01	0.03 to 0.17	0.09	0.12	.48	-0.15 to 0.32
**Household car ownership**									
	Did not own car(s)	Reference				Reference			
	Owned car(s)	-0.03	0.03	.32	-0.09 to 0.03	0.13	0.10	.22	-0.08 to 0.33
**Tobacco use status**									
	Not at all	Reference				Reference			
	Less than daily	0.01	0.03	.85	-0.06 to 0.73	-0.10	0.11	.37	-0.32 to 0.12
	Daily	-0.01	0.04	.78	-0.08 to 0.06	0.19	0.13	.14	-0.06 to 0.44
Location		-0.03	0.05	.60	-0.12 to 0.07	N/A	N/A	N/A	N/A
Companionship		-0.02	0.04	.66	-0.10 to 0.06	N/A	N/A	N/A	N/A
Self-reported tobacco use		0.04	0.09	.65	-0.14 to 0.23	-0.24	0.19	.20	-0.62 to 0.13
Saw other people using tobacco		0.23	0.10	.02	0.03 to 0.43	-0.20	0.23	.40	-0.65 to 0.26
Saw evidence of using tobacco		0	0.08	.99	-0.17 to 0.17	0.09	0.23	.69	-0.36 to 0.55
Smelled tobacco use		-0.11	0.06	.07	-0.23 to 0.01	0.10	0.22	.65	-0.34 to 0.54
Saw protobacco messages		0.01	0.08	.88	-0.15 to 0.18	0.07	0.19	.77	-0.44 to 0.32
Saw antitobacco messages		-0.17	0.07	.01	-0.31 to -0.04	-0.06	0.32	.77	-0.44 to 0.32

^a^ *R* ^2^ of this model is .24, adjusted *R* ^2^ is .17 (F_16,171_=3.4, n=188).

^b^ *R* ^2^ of this model is .11, adjusted *R* ^2^ is .03 (F_14,167_=1.4, n=182).

^c^Not applicable (N/A).

^d^ *P* values in italics are significant.

#### Validity of Ecological Momentary Assessment Data

Tables 2 and 3 offer pairwise correlations for 5-day and 10-day assessments, respectively. For each pair of compliance outcomes, the correlations between momentary assessment and end-of-day assessment were significant. The correlation coefficient remains the highest for self-reported tobacco use (*r*=.54 and .55, *P*<.001), followed by seeing protobacco messages (*r*=.49, *P*<.001; *r*=.50, *P*<.001), and seeing antitobacco messages (*r*=.49, *P*<.001; *r*=.39, *P*<.001). The correlation coefficients varied slightly for compliance measurements when compared to the 5-day and 10-day assessments, indicating that momentary and end-of-day compliance level was steady during the 10-day study period.

**Table 2:** The zero-order correlation of tobacco-related variables between momentary and end-of-day surveys, with assessment at study day 5.

Variable item	Momentary assessment, mean (SD)	End-of-day assessment, mean (SD)	Correlation, *r*	*P*
Used tobacco product(s)	.08 (.20)	.17 (.31)	.54	<.001
Saw other people smoking	.13 (.31)	.45 (.39)	.23	<.001
Saw evidence of smoking	.17 (.35)	.39 (.38)	.36	<.001
Smelled tobacco	.26 (.40)	.41 (.38)	.21	<.001
Saw protobacco messages	.15 (.25)	.08 (.21)	.49	<.001
Saw antitobacco messages	.25 (.30)	.28 (.35)	.40	<.001

**Table 3:** The zero-order correlation of tobacco-related variables between momentary and end-of-day surveys, with assessment at study day 10.

Variable item	Momentary assessment, mean (SD)	End-of-day assessment, mean (SD)	Correlation, *r*	*P*
Used tobacco product(s)	.08 (.20)	.17 (.30)	.55	<.001
Saw other people smoking	.13 (.32)	.44 (.37)	.23	<.001
Saw evidence of smoking	.17 (.36)	.38 (.37)	.34	.002
Smelled tobacco	.27 (.43)	.39 (.36)	.17	.02
Saw protobacco messages	.15 (.25)	.08 (.18)	.50	<.001
Saw antitobacco messages	.25 (.30)	.27 (.33)	.39	<.001

## Discussion

### Principal Findings

This project successfully used the EMA approach to collect data in the Indian cities of Kolkata and Hyderabad. Like EMA research done in the United States and other middle- to high-income countries, the approach was used among people of different demographics and smoking statuses [22]. While alterations can improve the quality of the data, this work shows that EMA is feasible in a low- and middle-income country.

In this study, only employment status predicted different EMA compliance rates and time to complete end-of-day surveys. Much of the sample’s unemployed group included students. Possibly, these participants were more frequently in locations where it was less appropriate to use a mobile phone and were, therefore, less likely to comply. Future studies may benefit from identifying key time periods during the day when participants are most willing and able to use their phones, such as during lunch or break time, or on weekends. Additional formative research, such as focus groups, may also increase compliance by helping researchers better understand reasons why a person may or may not respond to EMA survey prompts.

We observed lower completion rates for momentary surveys than end-of-day surveys. This finding may be due to lower burden and randomness of survey prompting—participants expected end-of-day surveys daily at 10pm, while they received momentary surveys multiple times at random during active work and school hours. Yet, momentary and end-of-day completion rates were highly related. End-of-day compliance appeared to be the most significant predictor for momentary compliance. Likewise, momentary compliance appeared to be the only significant predictor for end-of-day compliance. This suggests that if one complies with momentary prompts, then he or she will also comply with end-of-day prompts. Another explanation for the disparity between momentary and end-of-day survey compliance is that the end-of-day surveys required less training and were, therefore, inherently easier to complete and were a lower burden. Future EMA studies could raise compliance with momentary prompts by increasing the number of study visits, compliance checks, or opportunities for retraining with participants before and during the data collection period.

### Limitations

This study had several limitations. First, we did not examine the trend of momentary and end-of-day compliance across the study period. It would be valuable to analyze whether participant compliance changes over time, and future studies may benefit from using longitudinal data analysis methods, such as the generalized estimating equation (GEE) model, to explore trends. Additionally, the predictors used in the EMA and EOD compliance models only explained 14% and 3% of variance in compliance outcomes, respectively. Although previous EMA studies found low prediction power [22,23], future studies need to test a wider range of predictors (eg, time of day, and day of the week) or other psychologically relevant factors (eg, positive and negative affects), which could explain more of the variation in EMA compliance. Further, the EMA app did not retain data from partial or expired responses to momentary or end-of-day survey prompts, or display whether participants employed the snooze function, which would provide valuable information on individual behavior and factors that explain completion. The snooze feature allowed participants to delay starting a survey—the intention was to increase the chance that a participant could complete a survey and thereby increase compliance. It is possible that participants could have turned off their phones at any point if they did not want to be disturbed, and there was no way of knowing from the data if or when that occurred. In future studies, researchers could set up time blocks with participants as part of the study training to establish when to keep the phone turned on, for example, by programming the app to only prompt them during the times they prefer.

The self-reported end-of-day survey data was used to confirm the validity of EMA assessment, instead of a biomedical measure for testing tobacco exposure. Future studies could combine momentary assessments with biochemically verified assessments, such as carbon monoxide (CO) monitors, and hair, saliva, or urine samples collected through additional study visits. This approach has been used in several US-based studies, including one of Southern California high school students’ physical activity, which paired mobile EMA data with heart rate and accelerometer data [24]. Another study of cocaine-addicted adults’ cravings and use paired mobile EMA with urine samples [25].

We sampled only in urban areas and did not randomly sample within these areas. In this vein, the enrollment criterion of owning an Android-capable mobile phone was a limitation, since it effectively excluded lower-tech phones and, consequently, a lower socioeconomic (SES) demographic. It is worth noting, however, that inexpensive “bootleg” versions of mobile phones and Android phones were widespread in our sample and study sites. Thus, this limitation may be relatively minor. Regardless, as mobile phones are increasingly used in EMA research, it is important to consider optimizing EMA software or apps for older mobile phone platforms. Alternately, issuing participants a mobile device for EMA studies may yield a more generalizable sample, particularly as older models of mobile phones become less expensive and easier to employ in research. However, it is important to consider that this research was part of a pilot study exploring relationships between demographics, tobacco exposure, and tobacco use in real time and natural settings using mobile phone EMA. In this paper, we specifically examined relationships of EMA compliance with various participant characteristics and behaviors. Thus, we did not intend to have a sample of participants that was nationally representative. Nonetheless, future studies could explore EMA compliance with a more generalizable sample of the population.

### Practice Implications

Overall, the participant completion rate of 46% in this study was lower than what has been observed in tobacco EMA studies performed in the United States and other developed countries, where rates ranged from 65% to 92% [9,26,27]. Researchers Stone and Shiffman suggest an 80% completion rate as a good measure of validity and generalizability of EMA data [28]. Our 72% end-of-day completion rate finding is promising. The end-of-day surveys were easier to employ in the app, and the higher completion rate than that of the momentary surveys suggests that the participants also experienced an easier time with these surveys. The literature supports brief recollection, such as a 24-hour recall period, as a legitimate EMA approach [3]. While end-of day assessment may risk capturing less of the momentary environment and experience [3], a lower-burden protocol may be better suited for more challenging populations and settings. Indeed, other mobile phone EMA research studies used a range of monitoring schemes that yielded high compliance, such as collecting data for one week or less [24,29,30], prompting participants to take a survey less than five times per day [30-32], or setting time blocks during the day in which participants will or will not be prompted [31]. Similar tobacco media exposure studies, such as that of Shadel and colleagues [9], used combined event- and time-based sampling. Adding in a component of user-initiated entry of tobacco exposure could increase completion rates of surveys and enrich the data.

It would be valuable to better understand expired or incomplete prompts. This study did not collect information on which of the prompts involved the snooze function before expiring. A possible extension of snooze time might result in more completed prompts. Additionally, the EMA app could be improved by allowing partial data from incomplete prompts to be visible because (1) data from those completed questions could still be extracted and analyzed, and (2) researchers could see whether participants tried to at least answer one question before letting the prompt expire, or if they simply did not respond. If most partially completed prompts had a certain number of the questions answered before expiring, it may indicate that the survey was too long.

We recommend repeated testing and fine tuning of an EMA protocol and technology to ensure protocol accuracy in future EMA studies, particularly when researchers build their own data collection app or software as was done in this study. The EMA field consultants interviewed during the planning phase reported using existing software services to collect and manage a dataset in their projects (personal communications by S Shiffman and M Rich, August 2012), but building a system from the ground up allowed more opportunity for customization and improvements. Additionally, we worked with local Indian developers to create the apps, endorsing the community-based research approach. Teaming up with local partners proved valuable for executing the EMA protocol and app development, as there were significant language and cultural barriers.

### Conclusions

This study employed EMA methodology in India, a low- and middle-income country with high tobacco use prevalence, and provided rich and instrumental evidence around the feasibility of using EMA measurements to capture real-time tobacco-related behaviors and participant compliance with a rigorous monitoring schedule. To our knowledge, this was the first study to examine compliance of an EMA study of tobacco use and tobacco-related cues in India or any LMIC. It is also the first EMA of a tobacco study that used an app that was integrated into participants’ personal mobile phones, as opposed to providing a separate mobile device for data collection. This paper may serve as a guide to other researchers interested in conducting EMA studies in LMIC, but the formative research and procedures must be customized to the specific health behavior and country of interest. Repeated testing of the protocol and software is particularly crucial to studies in foreign settings for two reasons: first, to troubleshoot for technical problems in the data collection and delivery system and, second, to ensure that concepts and messages are not lost or misunderstood in translation or culture between researcher and participant.

Recommendations for future studies using a mobile phone EMA include adapting instruments for lower SES populations and developing data collection platforms for basic-feature mobile phones. While it is possible to use momentary prompts, this work suggests the less burdensome approach of end-of-day surveys may be better. Future work should continue to explore methodological approaches, especially as mobile technology access becomes more universal.

## Appendix

Multimedia Appendix 1 A copy of the questions employed in the momentary and end-of-day surveys in the ecological momentary assessment (EMA) mobile app.

## Abbreviations

CO: carbon monoxide
EMA: ecological momentary assessment
EOD: end of day
GATS: Global Adult Tobacco Survey
GEE: generalized estimating equation
IQR: interquartile range
IRB: Institutional Review Board
LMIC: low- and middle-income countries
N/A: not applicable
PDA: personal digital assistant
SES: socioeconomic
WHO: World Health Organization
