# Supplementary material for: Using Ecological Momentary Assessment to Study Tobacco Behavior in Urban India: There’s an App for That
Source: JMIR Res Protoc. 2015 Jun 24;4(2):e76. doi: 10.2196/resprot.4408 (PMC4526962; doi:10.2196/resprot.4408)
Supplement: Multimedia Appendix 1 [file resprot_v4i2e76_app1.pdf]

MULTIMEDIA APPENDIX 1

EMA Mobile App: Momentary Prompt

We are going to ask you some questions about your surroundings. Answer as best you can.
[Press OK to start]

1. Where are you?
   - i. Home
   - ii. Work-place
   - iii. Other's home
   - iv. Bar/restaurant
   - v. Vehicle
   - vi. Outside
   - vii. Place of worship?
   - viii. Store/shopping place
   - ix. Other

2. Are you with someone?
   - i. No, I am alone
   - ii. Yes
     1. With my spouse
     2. With family members/ relatives
     3. With friends
     4. With co-worker(s)
     5. With people I don't know

3. Are you currently using a tobacco product?
   - i. No
   - ii. Yes, Smoke tobacco
     1. Manufactured/branded cigarette
     2. Rolled tobacco in paper or leaf
     3. Bidi
     4. Cigar, cheroots, or cigarillo
     5. Hookah or water pipe
   - iii. Yes, Smokeless tobacco
     1. Betel quid with tobacco
     2. Khaini or tobacco lime mixture
     3. Gutkha or tobacco lime, areca nut mixture
     4. Oral tobacco such as snuff, mishri, qul, gudakhu

4. Are other people using tobacco products in your current location?
   - i. No
   - ii. Yes, in my group
     1. Using smoke tobacco
     2. Using smokeless tobacco
     3. Both
   - iii. Yes, in view
     1. Using smoke tobacco
     2. Using smokeless tobacco
     3. Both

5. Do you see evidence of tobacco use around your current location? If yes, check all that you see.

- i. No, I do not see anything
- ii. Yes
  - 1. Cigarette butts
  - 2. Bidi butts
  - 3. Ashtrays (used only)
  - 4. Spit from oral tobacco (such as snuff, mishri, betel quid)

6. Can you **smell** tobacco smoke in your current location?

- i. No
- ii. Yes

7. Is smoking allowed in your current location?

- i. Allowed
- ii. Not allowed, but with exceptions
- iii. Never allowed
- iv. No Rules
- v. Don't know

8. Is smokeless tobacco use allowed in your current location?

- i. Allowed
- ii. Not allowed, but with exceptions
- iii. Never allowed
- iv. No Rules
- v. Don't know

Thank you for answering the questions about your surroundings. Now we are going to ask you some questions about your media. Answer as best you can.
[Press OK to start.]

9. In your current location, do you see any information in **newspapers or magazines** about the dangers of using tobacco products or that encourage quitting use of tobacco products?

- i. No
- ii. Yes
  - Check all products that are covered:
  - 1. Cigarettes
  - 2. Bidis
  - 3. Smokeless tobacco

10. In your current location, do you see any information on **television** about the dangers of using tobacco products or that encourage quitting use of tobacco products?

- i. No
- ii. Yes
  - Check all products that are covered:
  - 1. Cigarettes
  - 2. Bidis
  - 3. Smokeless tobacco

11. In your current location, do you hear any information on the **radio** about the dangers of using tobacco products or that encourage quitting use of tobacco products?

i. No
ii. Yes
Check all products that are covered:
1. Cigarettes
2. Bidis
3. Smokeless tobacco

12. In your current location, do you see any information on **billboards or posters** about the dangers of using tobacco products or that encourage quitting use of tobacco products?

i. No
ii. Yes
Check all products that are covered:
1. Cigarettes
2. Bidis
3. Smokeless tobacco

13. In your current location, do you see any health warning labels on **cigarette packages**?

i. No
ii. Yes

14. In your current location, do you see any health warning labels on **smokeless tobacco packages**?

i. No
ii. Yes

15. In your current location, do you see any advertisements or signs in **newspapers or magazines** promoting tobacco products?

i. No
ii. Yes
Check all products that are covered:
1. Cigarettes
2. Bidis
3. Smokeless tobacco

16. In your current location, do you see any advertisements or signs on **television** promoting tobacco products?

i. No
ii. Yes
Check all products that are covered:
1. Cigarettes
2. Bidis
3. Smokeless tobacco

17. In your current location, do you hear any advertisements or signs on the **radio** promoting tobacco products?

i. No
ii. Yes
Check all products that are covered:
1. Cigarettes

2. Bidis
3. Smokeless tobacco

18. In your current location, do you see any advertisements or signs on **billboards or posters** promoting tobacco products?

i. No

ii. Yes

Check all products that are covered:

1. Cigarettes
2. Bidis
3. Smokeless tobacco

Thank you for completing the survey. Please continue to complete the surveys promptly.

APPENDIX B

EMA Mobile App: End-of-Day Prompt

We are going to ask you some questions about your day. Answer as best you can. You may estimate if you cannot recall exactly.
[Press OK to start.]

1. Did you use any tobacco products **today**?
    - i. No
    - ii. Yes, Smoke tobacco
        1. Manufactured/branded cigarette
        2. Rolled tobacco in paper or leaf
        3. Bidi
        4. Cigar, cheroots, or cigarillo
        5. Hookah or water pipe
    - iii. Yes, Smokeless tobacco
        1. Betel quid with tobacco
        2. Khaini or tobacco lime mixture
        3. Gutkha or tobacco lime, areca nut mixture
        4. Oral tobacco such as snuff, mishri, qul, gudakhu

    About how many times did you use tobacco today?
    1. Only 1 time
    2. 2 to 5 times
    3. 6 to 10 times
    4. 11 to 15 times
    5. 15 to 20 times
    6. More than 20 times

2. Did you **see** other people using tobacco today?
    - i. No
    - ii. Yes

    Who were the people you saw smoking today? You can select more than one option.
    1. Spouse
    2. Friends
    3. Family/relatives
    4. Co-workers
    5. People nearby that I do not know

3. Did you **see** any evidence of tobacco use today?
    - i. No **[skips to question 4]**
    - ii. Yes

    Check all that you saw.
    1. Cigarette butts
    2. Bidi butts
    3. Ashtrays (used only)
    4. Spit from oral tobacco (such as snuff, mishri, betel quid)

4. Did you **smell** tobacco smoke at any time today?
i. No
ii. Yes

5. Did you **see** or **hear** any messages today that said it was good to use tobacco?
i. No **[skips to 6]**
ii. Yes

Check all of the places you saw or heard messages that said it was good to use tobacco products.
1. Television show (including popular characters using tobacco)
2. Print story or ad in a newspaper or magazine
3. Radio story or advertisement
4. Billboard poster (including advertisements) in the street
5. Billboard or poster (including advertisements) near or within a store
6. Packaging of tobacco products
7. Movie theater

6. Did you see or hear any messages today that said it was bad to use tobacco?
i. No **[skips to 7]**
ii. Yes
Check all of the places you saw or heard a message saying it was bad to use tobacco products.
1. Television show (including popular characters or news reports)
2. Print story or ad in a newspaper or magazine
3 Radio story or advertisement
4. Billboard poster (including advertisements) in the street
5. Billboard or poster (including advertisements) near or within a store
6. Packaging of tobacco products
7. Movie theater

7. Overall, was the total number of tobacco messages you saw or heard today like other days?
i. Number of tobacco messages was **higher** than a typical day
ii. Number of tobacco messages was **lower** than a typical day
iii. Number of tobacco messages was **as typical** as most days
iv. Not sure

8. Were there any special messages that you can recall seeing or hearing about tobacco today?
i. No
ii. Yes
1. What was the message? **[Fill-in space for explanation]**
2. Where did you see/hear the message**? [Fill-in space for explanation]**
